# *Bacteroides fragilis* Toxin Induces Intestinal Epithelial Cell Secretion of Interleukin-8 by the E-Cadherin/β-Catenin/NF-κB Dependent Pathway

**DOI:** 10.3390/biomedicines10040827

**Published:** 2022-03-31

**Authors:** Chang-Gun Lee, Soonjae Hwang, Sun-Yeong Gwon, Chanoh Park, Minjeong Jo, Ju-Eun Hong, Ki-Jong Rhee

**Affiliations:** 1 Department of Biomedical Science, College of Software and Digital Healthcare Convergence, Yonsei University MIRAE Campus, Wonju 26493, Korea; dangsunsang@ajou.ac.kr (C.-G.L.); soonjaehwang91@gmail.com (S.H.); sunyeong.gwon@kbri.re.kr (S.-Y.G.); cksdh9453@gmail.com (C.P.); minjeongjo@yonsei.ac.kr (M.J.); jehong@yonsei.ac.kr (J.-E.H.); 2 Department of Medical Genetics, School of Medicine, Ajou University, Suwon 16499, Korea; 3 Department of Biochemistry, Lee Gil Ya Cancer and Diabetes Institute, GAIST, College of Medicine, Gachon University, Incheon 21999, Korea; 4 Neural Circuits Group, Korea Brain Research Institute, Daegu 41062, Korea

**Keywords:** enterotoxigenic *Bacteroides fragilis*, colonic epithelial cell, E-cadherin, β-catenin, NF-κB

## Abstract

Enterotoxigenic *Bacteroides fragilis* (ETBF) has emerged as a gut microbiome pathogen that can promote colitis associated cancer in humans. ETBF secretes the metalloprotease, *B. fragilis* toxin (BFT), which can induce ectodomain cleavage of E-cadherin and IL-8 secretion through the β-catenin, NF-κB, and MAPK pathways in intestinal epithelial cells. However, it is still unclear whether E-cadherin cleavage is required for BFT induced IL-8 secretion and the relative contribution of these signaling pathways to IL-8 secretion. Using siRNA knockdown and CRISPR knockout studies, we found that E-cadherin cleavage is required for BFT mediated IL-8 secretion. In addition, genetic ablation of β-catenin indicates that β-catenin is required for the BFT induced increase in transcriptional activity of NF-κB, p65 nuclear localization and early IL-8 secretion. These results suggest that BFT induced β-catenin signaling is upstream of NF-κB activation. However, despite β-catenin gene disruption, BFT still activated the MAPK pathway, suggesting that the BFT induced activation of the MAPK signaling pathway is independent from the E-cadherin/β-catenin/NF-κB pathway. These findings show that E-cadherin and β-catenin play a critical role in acute inflammation following ETBF infection through the inflammatory response to BFT in intestinal epithelial cells.

## 1. Introduction

Enterotoxigenic *Bacteroides fragilis* (ETBF) is an obligate anaerobic bacterium that causes diarrheal disease in livestock and humans [1,2]. Experimental studies have demonstrated that ETBF associated diarrhea is associated with histologic disruption of the intestinal barrier and inflammation [3,4]. In addition, several studies indicate that ETBF promotes ulcerative colitis, Crohn’s disease and colon cancer [5,6]. The most well characterized virulence factor of ETBF is the secreted 20-kDa heat-labile metalloproteinase, called *B. fragilis* toxin (BFT) [7,8]. BFT induces the disruption of intestinal epithelial cell integrity through ectodomain cleavage of the cellular junctional protein E-cadherin [9]. E-cadherin cleavage is associated with IL-8 secretion in intestinal epithelial cells, resulting in the recruitment of immune cells to the colonic mucosa [10,11].

BFT contains a zinc-binding metalloprotease motif (HEXXHXXGXXH) that is critical for its biological activity [12]. Recombinant strains of *B. fragilis* (rNTBF) secreting a catalytically inactive form of BFT (such as H352) lose their biologic activity, resulting in the inhibition of E-cadherin cleavage, and IL-8 secretion in intestinal epithelial cells [13]. The cytoplasmic domain of E-cadherin interacts with β-catenin and α-catenin, forming the E-cadherin-catenin complex [14,15]. The E-cadherin-catenin complex binds with the cellular actin cytoskeleton scaffold, maintaining cell morphology and modulating signal transactivation of β-catenin [16,17]. β-catenin regulates a variety of cellular processes, such as cell proliferation, differentiation, and inflammatory responses [18]. Especially, β-catenin plays a major role in triggering colonic inflammatory responses in colitis. β-catenin, in turn, is regulated by the adenomatous polyposis coli (APC) protein, axin, and glycogen synthase kinase-3β (APC/axin/GSK-3β) complex [19]. Loss of E-cadherin can lead to the release of free cytosolic β-catenin, which leads to β-catenin nuclear translocation, triggering Wnt/β-catenin signaling [20].

The role of the E-cadherin/β-catenin complex in BFT mediated inflammation has been previously described in the human epithelial cell line HT29/C1 [21]. In HT29/C1 cells, BFT induces a sequential cell signaling cascade: E-cadherin ectodomain cleavage, γ-secretase-dependent juxtamembrane cleavage, β-catenin nuclear translocation, and IL-8 secretion [22]. BFT mediated β-catenin activation has also been demonstrated in animal studies, where Min^Apc+/−^ mice were infected with ETBF. Min^Apc+/−^ mice have enhanced activation of the β-catenin pathway due to heterozygous mutation in an Apc allele. This leads to increased tumorigenesis due to the further enhancement of β-catenin activation [23]. In addition to E-cadherin cleavage/β-catenin nuclear translocation, BFT has been reported to induce other cellular signaling pathways such as nuclear factor-kappa B (NF-κB) and mitogen activated protein kinase (MAPK) pathways in intestinal epithelial cells, resulting in the subsequent secretion of IL-8 [24,25]. These signaling pathways are also upregulated in the mouse intestinal epithelium of ETBF colonized mice, further providing evidence that BFT is a major factor in ETBF mediated colitis and colitis associated cancer [26]. Taken together, several studies have demonstrated that ectodomain cleavage of E-cadherin stimulates the NF-κB and MAPK pathways [27,28,29,30], as well as the β-catenin pathway, which leads to IL-8 secretion [31,32]. However, several aspects of BFT induced IL-8 secretion are unclear. First, it is known that BFT induces rapid E-cadherin ectodomain cleavage; however, it is unknown whether E-cadherin ectodomain cleavage is required for BFT induced IL-8 secretion. Second, it is known that BFT triggers β-catenin, NF-κB, and MAPK signaling; however, it is unknown whether all of these pathways contribute to subsequent IL-8 secretion. Finally, if these three pathways do contribute to IL-8 secretion, it is unclear whether a sequential activation of these three pathways occur in response to BFT.

In this study, we examined the role of the E-cadherin/β-catenin complex in BFT induced inflammatory signaling in the colonic epithelial cell line, HT29/C1. BFT induction of IL-8 secretion was examined in cells deficient in the E-cadherin or β-catenin pathway. The role of NF-κB in the context of the β-catenin pathway was also examined. Our data suggests that BFT induced IL-8 secretion requires the presence of E-cadherin and that the activation of the β-catenin pathway occurs upstream of the NF-κB pathway. Additionally, BFT activation of the MAPK pathway appears to be independent of the β-catenin pathway. These data provide a mechanistic understanding of BFT effects on cells and implicate multiple pathways for IL-8 secretion by BFT.

## 2. Materials and Methods

### 2.1. Cell Culture and Cell Fractionation

Human colonic epithelial HT29/C1 cells were incubated in Dulbecco’s modified Eagle’s medium (Invitrogen, Carlsbad, CA, USA) supplemented with 10% fetal bovine serum (Invitrogen, Carlsbad, CA, USA), 25 mM HEPES and penicillin (100 U/mL)/streptomycin (100 μg/mL). To enumerate viable cells, cells were seeded with a density of 2 × 10^5^ cells/mL in 60 mm culture plates and cultured for 10 days. Cells were detached with 0.25% trypsin-EDTA (Invitrogen, Carlsbad, CA, USA) and neutralized with DMEM containing 10% FBS. Unstained cells (viable) were counted on a hemocytometer (Marienfeld, Lauda-Königshofen, Germany) under a microscope. Each experiment was carried out in triplicate and the results were expressed as mean ± standard error of mean (SEM) for each treatment group. For subcellular fraction, cells were maintained at 37 °C in a cell culture incubator with 5% CO_2_. Cells were harvested with 400 μL of hypotonic solution buffer (20 mM Tris-HCl, 3 mM MgCl_2_ and 10 mM NaCl) with a protease inhibitor cocktail (Roche Diagnostics, Mannheim, Germany). The cells were incubated for 10 min on ice and homogenized 40 times with a Dounce homogenizer (Wheaton, Millville, NJ, USA). The cell lysates were centrifuged at 4 °C for 10 min at 5000× *g*, and the supernatant (cytoplasmic fraction) was transferred to a new microfuge tube and mixed with 5X SDS-loading buffer. The pellet (nuclear fraction) was washed once with hypotonic solution buffer and lysed with 5X SDS-loading buffer. Before Western blotting, nuclear fraction samples were sonicated for 5 s at 20 Hz (Vibracell, Danbury, CT, USA).

### 2.2. Bacterial Strains and Bacterial Supernatant

Nontoxigenic wild type *B. fragilis* (WT-NTBF) overexpressing an active form of BFT (rETBF; *bft*-2) and WT-NTBF overexpressing a biologically inactive mutated *bft* (rNTBF; *bft*-2 H352Y) were a generous gift from Cynthia Sears and Augusto Franco (Johns Hopkins University, Baltimore, MA, USA). All *Bacteroides* strains used in this study are naturally resistant to gentamicin and transformed with pFD340 conferring clindamycin resistance. Both recombinant *Bacteroides* strains (rETBF, rNTBF) were grown in brain heart infusion (BHI) agar supplemented with hemin, vitamin K1, cysteine, and two antibiotics (clindamycin and gentamicin) at 37 °C under anaerobic conditions (Pack-Anaero, Mitsubishi Gas Chemical Co. Inc., New York, NY, USA) for two days. Thereafter, a single bacterial colony was picked and cultured in BHI broth at 37 °C under anaerobic condition for another two days. The bacterial supernatants were centrifuged, filtered through a 0.45 μm syringe filter (Satorius, Goettingen, Germany) and stored at −80 °C until used.

### 2.3. Small-Interference RNA (siRNA) Transfection

HT29/C1 cells were seeded in 24-well plates (2 × 10^5^ cells/well) in antibiotic and serum free DMEM and cultured for 2 h. Small interference RNA (siRNA) and transfection reagent (DharmaFECT 4, Dharmacon, Lafayette, CO, USA) were mixed and overlaid onto the cells overnight. Thereafter, 0.5 mL of DMEM medium supplemented with 10% FBS was added to each well and cultured for the indicated duration. E-cadherin siRNA (SMARTpool), scramble siRNA and transfection reagents were purchased from Dharmacon. For IL-1β treatment studies, we harvested E-cadherin siRNA transfected cells that detached from the cell plates, washed them once with serum free medium and reseeded into 24-well plates using serum free DMEM (5 × 10^5^ cells/well). Cells were immediately treated with recombinant IL-1β (10 ng/mL, R&D Systems, Minneapolis, MN, USA), rETBF supernatant (1:10 dilution), or rNTBF supernatant (1:10 dilution) for 6 h. The cell culture supernatant was harvested and stored at −80 °C until analyzed by IL-8 ELISA.

### 2.4. Western Blot Analysis

HT29/C1 cells were washed with PBS and lysed at 4 °C with radioimmunoprecipitation (RIPA) buffer (Sigma, St. Louis, MO, USA) containing a protease inhibitor cocktail (Roche Diagnostics, Mannheim, Germany) and phosphatase inhibitor cocktail (Sigma, St. Louis, MO, USA). The cell lysates were incubated on ice for 10 min and centrifuged at 12,000× *g* for 10 min at 4 °C. The supernatant protein was collected and quantified using the Bradford Protein assay (Bio-Rad, Hercules, CA, USA). Protein samples were separated using sodium dodecyl sulfate-polyacrylamide gel electrophoresis (SDS-PAGE) and transferred to nitrocellulose membranes (Pall, Washington, NY, USA). For measuring alterations in HT29/C1 cell proteins, we analyzed the lysates with the optimal concentrations of antibodies. A detailed description of the antibodies used in this study are described in the Supplementary Materials Table S1. The immune labeled proteins were visualized using enhanced chemiluminescence (ECL) kit (Bio-Rad, Hercules, CA, USA).

### 2.5. Generation of β-Catenin Knockout HT29/C1 Cells

Three plasmids (lentiCRISPR v2, pMD2.G, psPAX) were purchased from Addgene (Cambridge, MA, USA) and individually transformed into *E. coli* DH5α cells (Enzynomics, Daejeon, Korea) to generate β-catenin knockout cell lines. Single guide RNAs (sgRNA) for *CTNNB1* (the gene encoding β-catenin) was designed using CRISPOR (crispor.tefor.net). Two sgRNAs targeting the third exon of *CTNNB1* gene (transcript ID: OTTHUMT00000258210.2) were used. The following sequences were used: *CTNNB1* KO #1 sgRNA forward sequence 5′–CAC CGT CCC ACT AAT GTC CAG CGT T–3′ and reverse sequence 5′–AAA CAA CGC TGG ACA TTA GTG GGA C-3′, CTNNB1 KO #2 sgRNA forward sequence 5′–CAC CGT GAT GGT TCA GCC AAA CGC–3′ and reverse sequence 5′–AAA CGC GTT TGG CTG AAC CAT CAC–3′. Designed sgRNAs were purchased from Cosmogenetech (Seoul, Korea) and each forward and reverse oligonucleotide was annealed to make double stranded oligonucleotides by TaKaRa thermal cycler dice TP600 (TAKARA, Shiga, Japan). Double stranded oligonucleotides were inserted into lentiCRISPR v2 vector, which was cleaved by Esp3I [BsmBI] (Thermo Fisher Scientific, Waltham, MA, USA). *CTNNB1* gRNA-lentiCRISPR v2 vector was co-transfected with pMD2.G (Addgene, Cambridge, MA, USA) and psPAX2 (Addgene, Cambridge, MA, USA) into HEK293T cells to produce lentiviruses containing *CTNNB1* gRNA-lentiCRISPR v2. After transfection, virus containing media was harvested at 48 and 72 h after transfection and concentrated using Lenti-X™ concentrator (Clontech, Mountain View, CA, USA) in accordance with the manufacturer’s instructions. Concentrated viruses were used to infect with cells (HT29/C1) in cultured media for 24 h, and then the virus containing media was removed. Infected cells were then selected with puromycin (5 mg/mL, Thermo Scientific). All the experiments were conducted under the approval of the Institutional Biosafety Committee of Yonsei University MIRAE Campus (IBC No. 201908-P-004-01). For analyzing genomic DNA of β-catenin, *CTNNB1*-gRNA lentiCRISPR v2 containing HT29/C1 cells were harvested, and genomic DNA was isolated using phenol:chloroform:isoamyl alcohol (25:24:1). To confirm *CTNNB1* from genomic DNA, we proceeded with nested PCR. The broad primer sequences were forward 5′–GTA AGA GTA TTA TTT TTC ATT GCC–3′ and reverse 5′–CTG TAA TAC AGG AAT TCA GAA AC–3′, and the nested primer sequences were forward 5′–GTT GTG GTG AAG AAA AGA GAG–3′ and reverse 5′–GGG TAG ACA TTC TGA AAC TAC TCC–3′. After *CTNNB1* amplification was confirmed, genomic DNA sequences were determined by Cosmogentech (Seoul, Korea).

### 2.6. Immunofluorescence and Confocal Microscopy

Cells were grown on sterilized glass coverslips and fixed with 4% paraformaldehyde solution (BIOSESANG, Seongnam, Korea) at 4 °C for 10 min, washed with PBS, and permeabilized with 0.2% Triton X-100 at RT for 20 min. Regarding immunostaining, cells were blocked with 1% bovine serum albumin in PBS for 30 min and stained with a 1 μg of primary antibody in 1% BSA-PBS solution overnight at 4 °C. Then, the cells were labeled with 1:1000 dilution of the appropriate fluorochrome labeled secondary antibodies (Alexa-Fluor-488 or Alexa-Fluor-568 (Invitrogen, Carlsbad, CA, USA)) in PBS at RT for 2 h. Finally, cells were washed three times with PBS and mounted with mounting medium containing 4′,6-diamidino-2-phenylindole (DAPI) (VECTOR laboratories LTD, Peterborough, UK). Images were captured with Carl Zeiss LSM710 confocal microscope (Carl Zeiss, Oberkochen, Germany).

### 2.7. Luciferase Reporter Assay

Cells were seeded in 24-well plates and incubated for 24 h before transfection. Reporter constructs (NF-κB luciferase and Renilla luciferase plasmid) (Addgene, Cambridge, MA, USA) were transfected using Lipofectamine^®^ 2000 (Invitrogen, Carlsbad, CA, USA). After 48 h of transfection, the cells were incubated with BFT supernatant, TNF-α (R&D Systems, Minneapolis, MN, USA), recombinant IL-1β (R&D Systems, Minneapolis, MN, USA) or lipopolysaccharide (Sigma, St. Louis, MO, USA) for 24 h. Then, the reporter assay was conducted using the Dual-Luciferase^®^ reporter assay system (Promega, Madison, WI, USA) and the luciferase activity was determined using a GloMax^®^ 20/20 luminometer (Promega, Madison, WI, USA). Each luciferase activity was normalized with Renilla luciferase activity.

### 2.8. Cytokine Analysis

Cell supernatants were collected and stored at −80 °C until analysis. Cytokine levels of IL-8 were determined using a commercialized ELISA kit (R&D Systems, Minneapolis, MN, USA) following the manufacturer’s instructions.

### 2.9. Statistical Analysis

Data in the bar graphs are presented as mean ± SEM. All statistical analyses were performed using GraphPad Prism 7.0 software (GraphPad Software, San Diego, CA, USA). All data were analyzed using an unpaired *t*-test and *p* < 0.05 was considered to be statistically significant (* *p* < 0.05, ** *p* < 0.01 and *** *p* < 0.001).

## 3. Results

### 3.1. E-Cadherin Is Required in BFT induced IL-8 Secretion

**Figure 1:**
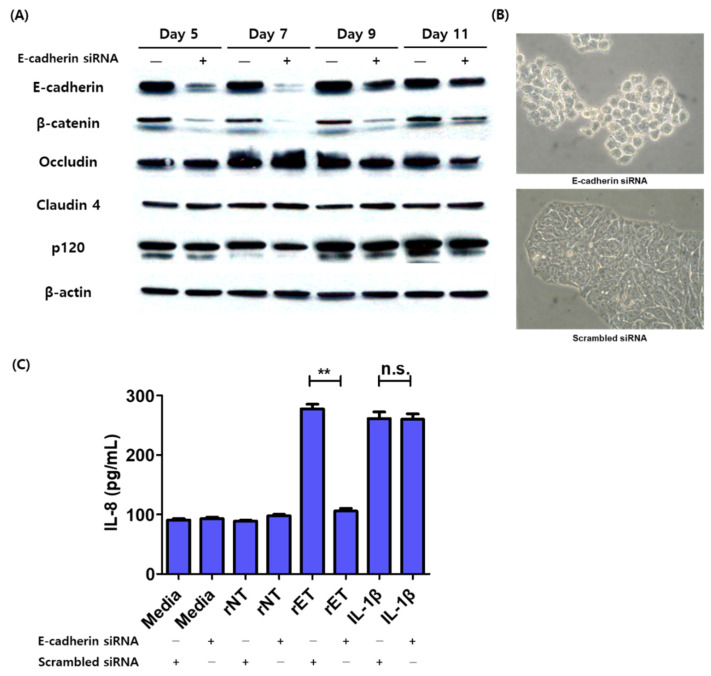
BFT induced IL-8 secretion is decreased in E-cadherin siRNA knockdown cells. (**A**) Western blot analysis of HT29/C1 cells treated with scrambled siRNA or E-cadherin siRNA for the indicated duration (5, 7, 9, or 11 days). β-actin was used as an internal control. (**B**) Cellular images of E-cadherin siRNA and scrambled siRNA transfected HT29/C1 cells (day seven). Representative images were captured using an inverted microscope (×200). (**C**) IL-8 ELISA of E-cadherin siRNA or scrambled siRNA transfected HT29/C1 cells incubated with rET and rNT (1:10 diluted with serum-free media) and recombinant IL-1β (10 ng/mL) for 6 h. Triplicate samples were analyzed. n.s., not significant; ** *p* < 0.01 (unpaired *t*-test).

To elucidate whether E-cadherin is required for BFT induced IL-8 secretion in colonic epithelial cells, we inhibited E-cadherin expression by siRNA knockdown. A time-course analysis of E-cadherin protein levels was conducted through Western blot analysis to determine the effectiveness of siRNA treatment and the optimal time point of E-cadherin knockdown. We found a dramatic decrease in E-cadherin protein levels around day 5–7 postknockdown, which then recovered gradually thereafter (Figure 1A). β-catenin protein levels also decreased in E-cadherin siRNA treated cells. Other cellular junctions, such as occludin, claudin 4, and p120 catenin, remained unchanged despite E-cadherin knockdown. E-cadherin siRNA treated HT29/C1 cells exhibited a cell rounding morphology, which demonstrates that the knockdown of E-cadherin leads to a physiologic change (Figure 1B). To determine whether BFT induced IL-8 secretion requires the presence of E-cadherin, we transfected cells with either E-cadherin siRNA or scrambled siRNA for 7 days, followed by treatment with recombinant ETBF supernatant (rET) or recombinant NTBF supernatant (rNT) for 6 h; IL-8 secretion was determined by ELISA. Cells lacking E-cadherin did not secrete IL-8, with levels almost comparable to nontreated controls (*p* < 0.005) (Figure 1C). The lack of E-cadherin did not compromise the ability to secrete IL-8 in response to other stimuli because treatment with recombinant IL-1β in E-cadherin siRNA knockdown cells induced similar levels of IL-8 secretion between scrambled siRNA-treated cells and E-cadherin siRNA-treated cells (Figure 1C). These results indicate that E-cadherin is required for BFT mediated IL-8 secretion in HT29/C1 cells.

### 3.2. Generation of CTNNB1 Knockout Colonic Epithelial Cells

**Figure 2:**
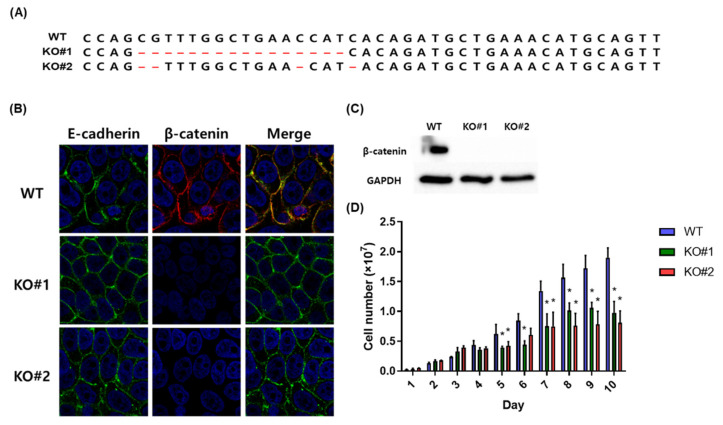
β-catenin gene disruption by CRISPR-Cas9 in HT29/C1 cells. (**A**) Validation of *CTNNB1* knockout in HT29/C1 cells. Genomic DNA of wild type (WT) and β-catenin knockout HT29/C1 cells (KO#1 and KO#2) were amplified by nested PCR and sequencing analysis of *CTNNB1* was conducted. The CRISPR-Cas9 system introduced an indel mutation (red) in the target sites of the *CTNNB1*. (**B**) Immunofluorescence imaging of WT, KO#1, and KO#2 cells. E-cadherin (green) and β-catenin (red). Nuclei were stained with DAPI (blue), and images were taken with a confocal laser-scanning microscope (×1640). (**C**) Western blot analysis of β-catenin in WT and β-catenin knockout HT29/C1 cells. GAPDH was used as an internal control. (**D**) Cellular proliferation of WT and β-catenin knockout HT29/C1 cells. Viable WT, KO#1 and KO#2 cells were enumerated by the trypan blue exclusion assay. * *p* < 0.05 vs. WT (unpaired *t*-test).

To investigate the role of β-catenin in the BFT induced inflammatory responses, we knocked out the β-catenin gene (*CTNNB1*) in HT29/C1 cells using the CRISPR-Cas9 gene-editing system. Two different guide RNAs for *CTNNB1* knockout were designed and cloned into a lentiviral vector. These *CTNNB1* knockout lentiviral vectors were packaged and named as lenti-*CTNNB1* KO#1 (KO#1) and lenti-*CTNNB1* KO#2 (KO#2). To confirm that the *CTNNB1* was disrupted, *CTNNB1* was examined by nested PCR and sequencing analysis in two gene edited independent cell clones to confirm that the *CTNNB1* gene was disrupted. In KO#1 and KO#2, 13 and 4 base pairs were deleted, respectively (Figure 2A). Immunofluorescence images (Figure 2B) and Western blotting analysis (Figure 2C) showed that β-catenin protein was absent, and the E-cadherin protein remained unaffected. In addition, we further examined whether disruption of *CTNNB1* affected cellular proliferation. Cellular proliferation of β-catenin knockout HT29/C1 (KO#1 and KO#2) cells was lower than that of wild type HT29/C1 cells (*p* < 0.05 for KO#1 and KO#2, Figure 2D). These results indicate that β-catenin was “functionally” eliminated by CRISPR-Cas9-mediated knockout.

### 3.3. BFT Does Not Induce IL-8 Secretion in β-Catenin Knockout Cells

**Figure 3:**
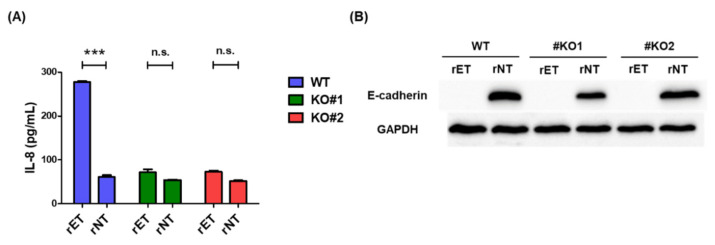
BFT does not induce IL-8 secretion in β-catenin knockout cells. (**A**) IL-8 secretion in BFT treated WT and β-catenin knockout (KO#1 and KO#2) HT29/C1 cells. WT, KO#1 and KO#2 cells were incubated with rET or rNT for 6 h. The cell supernatants were analyzed by IL-8 ELISA. The samples were analyzed in triplicate. n.s., not significant; *** *p* < 0.001 vs. WT (unpaired *t*-test). (**B**) Western blot analysis of E-cadherin in WT, KO#1, and KO#2 cells incubated with rET or rNT for 6 h. GAPDH was used as an internal control.

To determine if β-catenin is required in BFT induced IL-8 secretion, we incubated WT and β-catenin knockout cells (KO#1 and KO#2) with rET and rNT for 6 h, and IL-8 secretion was analyzed by ELISA. In contrast to WT HT29/C1 cells, IL-8 secretion was ablated in β-catenin knockout HT29/C1 cells treated with rET (*p* < 0.0001) (Figure 3A). In a previous study, we showed that E-cadherin cleavage is associated with BFT induced IL-8 secretion [13]. Therefore, we examined whether E-cadherin was cleaved in BFT treated β-catenin knockout cells. Our results indicated that E-cadherin was cleaved by BFT in β-catenin knockout cells (Figure 3B). Taken together, these results suggest that E-cadherin cleavage is not sufficient for the induction of IL-8 secretion, and the β-catenin pathway is required for BFT induced IL-8 secretion.

### 3.4. BFT Does Not Induce NF-κB Activation in β-catenin Knockout Cells

**Figure 4:**
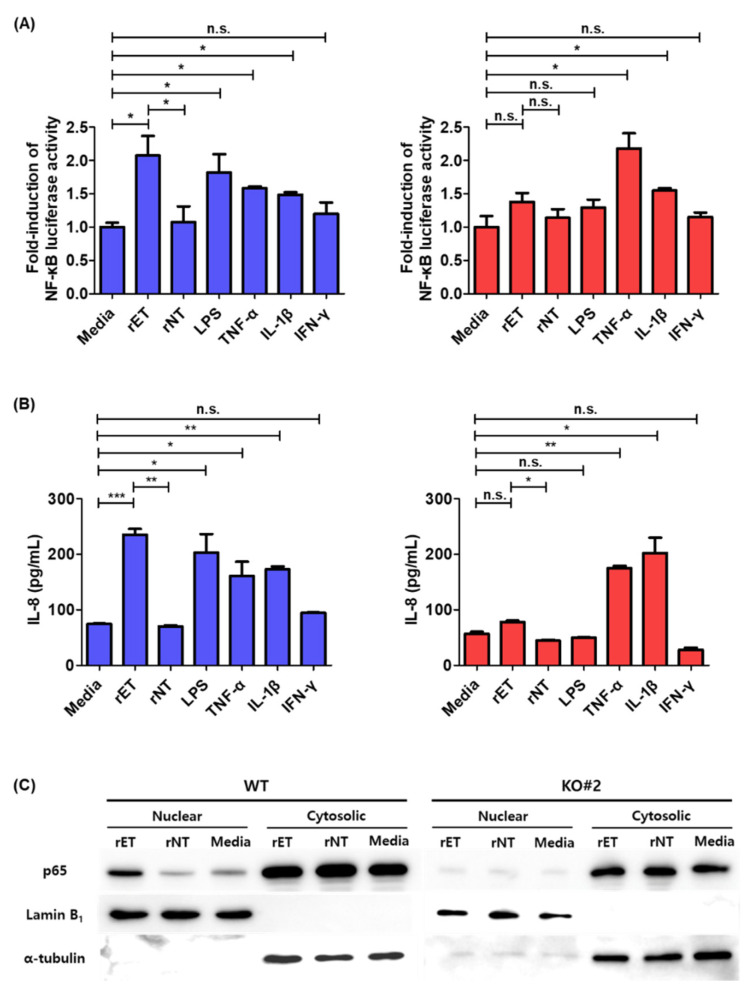
BFT does not increase NF-κB transcriptional activation in β-catenin knockout cells. (**A**) NF-κB luciferase activity of BFT incubated WT and β-catenin knockout (KO#2) HT29/C1 cells. WT (left) and KO#2 (right) cells were transfected with the NF-κB *Renilla* luciferase reporter for 48 h. Then, cells were incubated with rET or rNT and lipopolysaccharide (LPS, 100 ng/mL), TNF-α (100 ng/mL), IL-1β (100 ng/mL), or IFN-γ (100 ng/mL) for 6 h. Luciferase activity was normalized with *Renilla* luciferase activity, and relative values were presented. The experiments were conducted in triplicate. (**B**) IL-8 secretion of BFT stimulated WT and β-catenin knockout HT29/C1 cells transfected with a NF-κB luciferase reporter. WT (left) and KO#2 (right) cells were incubated with rET or rNT and LPS, TNF-α, IL-1β, or IFN-γ for 6 h. The cell supernatants were analyzed by IL-8 ELISA. The samples were analyzed in triplicate. (**C**) Western blot analysis of p65 NF-κB nuclear translocation in HT29/C1 cells. Subcellular fractions of WT and KO#2 cells incubated with rET or rNT for 6 h. Lamin B_1_ was used as an internal control for the nuclear fraction, and α-tubulin was used as an internal control for the cytosolic fraction. n.s. not significant; * *p* < 0.05; ** *p* < 0.01; *** *p* < 0.001 (unpaired *t*-test).

NF-κB transcriptional activity was analyzed through the luciferase reporter assay in β-catenin knockout cells to determine whether the NF-κB signaling is modulated by β-catenin in BFT treated cells. In contrast to WT HT29/C1 cells, β-catenin knockout cells showed no statistical increase in NF-κB transcriptional activity following BFT treatment (*p* < 0.05) (Figure 4A). This result indicates that NF-κB activation is downstream of β-catenin activation. Several molecules known to induce NF-κB activation (e.g., TNF-α) increased NF-κB transcriptional activity, showing that the NF-κB activation pathway was intact in β-catenin knockout cells. Generally, the IL-8 secretion data were consistent with the NF-κB luciferase data (Figure 4B). To confirm the NF-κB luciferase results, we also assessed the nuclear localization of p65, a hallmark of NF-κB activation, in BFT treated WT and β-catenin knockout cells through Western blot analysis. Our data show that BFT treatment induced the nuclear localization of p65 in WT cells but not in β-catenin knockout cells (Figure 4C). These results indicate that NF-κB activation and IL-8 secretion are generally intact in β-catenin knockout cells, but BFT induced NF-κB activation and IL-8 secretion requires intact β-catenin.

### 3.5. BFT induced Activation of the MAPK Pathway Is Partially Inhibited in β-Catenin Knockout Cells

**Figure 5:**
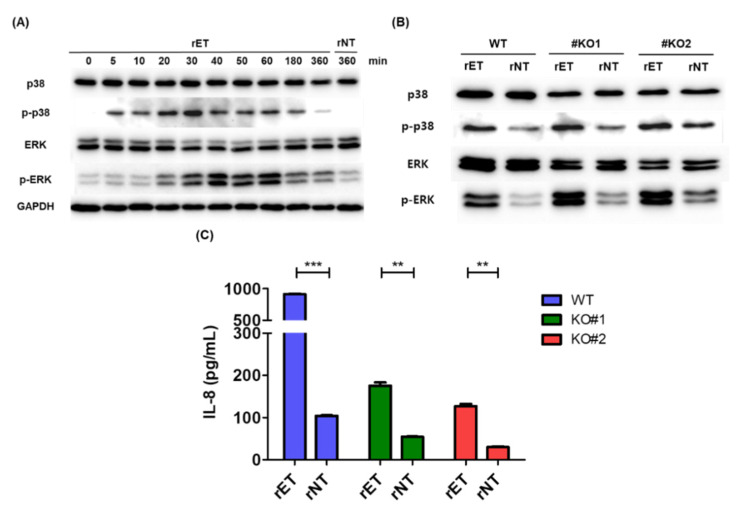
BFT induced MAPK activation does not require β-catenin. (**A**) Western blot analysis of MAPK signaling molecules. Wild type HT29/C1 cells were pre-incubated with serum free media for 12 h. Then cells were incubated with rET or rNT for the indicated time period (0, 5, 10, 20, 30, 40, 50, 60, 180, or 360 min). Cell lysates were subjected to Western blotting to quantify MAPK signaling molecules (p38, p-p38, ERK and p-ERK). GAPDH was used as an internal control. (**B**) Western blot analysis of MAPK proteins in BFT treated WT, KO#1, and KO#2 cells. WT, KO#1, and KO#2 cells were incubated with rET or rNT for 40 min. Cell lysates were subjected to Western blotting to evaluate MAPK signaling molecules. GAPDH was used as an internal control. (**C**) IL-8 ELISA. WT, KO#1, and KO#2 cells were incubated with rET or rNT for 24 h. Cell supernatants were analyzed through IL-8 ELISA. The samples were analyzed by triplicate and depicted as the mean. Error bars, SEM. ** *p* < 0.01, *** *p* < 0.001 (unpaired *t*-test).

We next examined whether BFT induced MAPK signaling was affected in β-catenin knockout cells. A time-course analysis of the MAPK activation pathway showed that activation of the MAPK proteins p38 and ERK was highest at approximately 30 and 40 min, respectively, after BFT treatment in WT HT29/C1 cells (Figure 5A). Therefore, we treated β-catenin knockout cells with either rET or rNT for 40 min and analyzed the levels of the activated phosphorylated p38 (p-p38) and phosphorylated ERK proteins (p-ERK). We found that, in BFT treated β-catenin knockout cells, rET treatment increased the levels of p-p38 and p-ERK comparable to that of WT cells (Figure 5B). This result suggests that BFT induces MAPK pathway activation in the absence of β-catenin, indicating that MAPK activation does not require β-catenin. In addition, treatment of cells with rET for 24 h showed that IL-8 secretion was increased in both WT (*p* < 0.0001) and in β-catenin knockout cells (*p* < 0.0001) (Figure 5C), although IL-8 secretion was drastically lower in β-catenin knockout cells compared to wild type cells (Figure 5C). Taken together, these results suggest that activation of the MAPK pathway is independent from that of the β-catenin pathway. However, the contribution of the MAPK pathway in BFT induced IL-8 secretion is lower compared to the β-catenin pathway. Additionally, it is possible that the β-catenin pathway is predominantly important during early IL-8 secretion (within 6 h), and that the MAPK pathway contributes to late IL-8 secretion (after 24 h).

## 4. Discussion

Colitis associated cancer is a type of colorectal cancer that is preceded by inflammation and can be observed in inflammatory bowel disease (IBD) patients [33]. In colorectal cancer patients, Wnt/β-catenin plays a key role in the early stage of tumorigenesis, which is also predominant in IBD patients [34]. In this regard, investigating the mechanisms by which inflammatory processes activate Wnt/β-catenin and consequent downstream signaling is important in understanding this disease. In addition, because E-cadherin directly binds to β-catenin, numerous studies implicated a positive correlation with E-cadherin dysregulation and Wnt/β-catenin signaling [35].

ETBF has emerged as a human enteric pathogen prominently contributing to IBD and colon tumorigenesis [36]. Recent studies on ETBF have shown that it is more likely to colonize the intestine than the non-enterotoxigenic *B. fragilis* (NTBF) of normal flora and acts as a tumor initiator by inducing colitis and DNA damage [37,38,39]. BFT was reported to enhance reactive oxygen species (ROS) and production, inducing DNA damage, and consequently activating lactate dehydrogenase (LDH) [40,41]. Therefore, monitoring BFT mediated LDH production through immunohistochemical analysis might be used to assess BFT mediated progression of colon tumorigenesis [42]. In addition, several probiotics that either prevent ETBF colonization and/or mitigate BFT effects may alleviate ETBF pathogenesis [43]. Since BFT is the key virulence factor of ETBF, elucidating how BFT affects intestinal cells is the first step to understanding ETBF induced inflammation and colon tumorigenesis.

Four key observations were made in the current study. First, BFT induced IL-8 secretion was dependent on the presence of E-cadherin. Second, BFT induced IL-8 secretion was dependent on the presence of β-catenin. Third, activation of the NF-κB pathway is dependent on and occurs downstream of β-catenin. Fourth, MAPK activation is partially dependent on the presence of β-catenin. It has been previously reported that the β-catenin/NF-κB/MAPK signaling pathways are activated in response to BFT treatment [44]. It was surmised that E-cadherin ectodomain cleavage is required to initiate the abovementioned signaling pathways. In addition, BFT induced IL-8 secretion was thought to occur via the β-catenin/NF-κB/MAPK pathways but the contribution of each individual pathway was unclear. Moreover, although a clear association between E-cadherin cleavage and IL-8 secretion was observed, the requirement of E-cadherin for IL-8 secretion was not tested definitively. Previously, we showed that the disruption of cell–cell E-cadherin interactions by EDTA treatment triggers IL-8 secretion [13]. In the current study, we showed that IL-8 secretion is absent in E-cadherin siRNA knockdown cells. This initially suggested that E-cadherin may be a candidate cognate receptor for BFT. However, the amino acid sequence of the putative E-cadherin cleavage site of BFT responsive cells and BFT nonresponsive cells indicates that BFT does not directly bind and cleave E-cadherin (data not shown). The cellular cognate receptor for BFT has yet to be determined.

E-cadherin plays an important role in physiologic cellular processes and cancer progression. Loss of E-cadherin is a well known feature of developmental epithelial–mesenchymal transition (EMT), and its loss in cancer plays a crucial role in the transition to malignant cancer [45]. In addition, proteolytic cleavage of E-cadherin produces protein fragments that possess oncogenic properties. In one study, it was reported that the cytoplasmic cleaved fragment of E-cadherin is translocated to the nucleus, activating the expression of Wnt related genes that are involved in cell proliferation and differentiation in vitro [46]. In addition, because the cytoplasmic domain of E-cadherin interacts with β-catenin, E-cadherin degradation releases membrane bound β-catenin, which, in turn, activates proliferation genes, such as cyclin D1, c-myc, and c-jun [47]. In this context, the determination of the cellular process of E-cadherin and β-catenin is important in understanding BFT induced effects in epithelial cells.

To elucidate the associations within BFT induced cellular signaling, the CRISPR-Cas9 gene editing system has the advantage of generating permanent, inherited knockout cell lines [48]. In this study, we examined the role of β-catenin in BFT induced inflammation, represented by IL-8 secretion. BFT rapidly cleaves E-cadherin, resulting in E-cadherin-β-catenin complex dissociation. β-catenin was targeted in several different cell lines using the CRISPR-Cas9 editing system [49,50], which has shown that the Wnt/β-catenin signaling plays a crucial role in colorectal cancer [51]. The β-catenin knockout HT29/C1 cell line developed in the current study should provide an additional tool for understanding Wnt/β-catenin signaling in colorectal cancer.

Activation of the NF-κB pathway was ablated in β-catenin knockout cells. However, activation of MAPK signaling was unaffected. Regarding IL-8 secretion, early IL-8 secretion (6 h) was diminished to control levels in β-catenin deficient cells. However, late IL-8 secretion (24 h) was greatly decreased but detectable in β-catenin deficient cells. Considering that the MAPK signaling pathway was activated in β-catenin deficient cells, it is possible that early IL-8 secretion is due to β-catenin activation, whereas late IL-8 secretion can be attributed to MAPK activation. Regardless, it is clear that the β-catenin pathway contributes overwhelmingly to BFT induced IL-8 secretion. Our data suggest that that two independent signaling pathways, namely, the β-catenin/NF-κB and MAPK pathways, are independently activated by BFT.

Our current study does have limitations. First, we examined the BFT mediated E-cadherin/β-catenin/NF-κB/IL-8 secretion signal cascade in a single colonic epithelial cell line, HT29/C1. Second, we did not elucidate the mechanisms of MAPK signaling induced by BFT. The independent activation of the β-catenin/NF-κB pathway and the MAPK pathway may be due to the presence of two different cognate receptors for BFT. Sears and colleagues proposed that the putative candidate receptor of BFT is the G protein coupled receptor 35 (GPR35). GPR35 is highly expressed in BFT responsive cells and the knockdown of GPR35 in HT29/C1 resulted in β-arrestin dependent loss of BFT induced effects [52]. Considering that β-arrestin is one of the major factors of the Wnt/β-catenin pathway [53], it is possible that GPR35 may act as BFT specific activator of Wnt/β-catenin in the colonic epithelium, consistent with our study.

In general, MAPK activation is associated with IL-8 secretion through the transcription factor AP-1 [54]. Consistent with this idea, Kim et al. reported that BFT induces MAPK signaling and IL-8 secretion through the activation of AP-1 [55]. Similar to our experimental approach, Kim et al. decreased β-catenin expression in HCT116 colon epithelial cells using RNA interference (RNAi) and found that BFT enhanced NF-κB transcriptional activity and IL-8 secretion [56]. The conflicting results may be due to the different cell lines used. More likely, the divergent result is due to the method of β-catenin ablation. RNAi based on technologies including small interfering RNA or short hairpin RNAs (shRNA), which reduces mRNA expression at the post-transcriptional level, were used extensively [57]. However, RNAi has the disadvantage of exerting transient effects lasting, at most, a few weeks [58]. Meanwhile, the CRISPR-Cas9 system has the advantage of generating permanent, inherited knockout cell lines, and, thus, is considered to be the next generation editing system. Our studies demonstrated a complete ablation of β-catenin using the CRISPR-Cas9 system and we could not detect the protein levels of β-catenin during continuous culture for a year (data not shown).

In the current study, we did not examine whether BFT induces late IL-8 secretion via AP-1 activation in β-catenin knockout cells. In addition, lithium chloride (LiCl) treatment would be helpful to understand the exact molecular pathways involved in BFT induced inflammatory signals, such as β-catenin, NF-κB and MAPK signaling. Similar to BFT, LiCl is known to activate the β-catenin, NF-κB, and MAPK pathways, resulting in IL-8 secretion [59,60], so determining whether LiCl activates NF-κB and MAPK in β-catenin knockout cells would be helpful to understand BFT mediated inflammatory signaling.

## 5. Conclusions

In summary, the results from this study demonstrate that β-catenin is required for BFT induced early IL-8 secretion and the subsequent nuclear translocation of NF-κB p65 in HT29/C1 cells. However, MAPK activation is independent of BFT induced β-catenin signaling. The results from this study outline several associations between BFT induced cellular mechanisms and help uncover the effects of ETBF on colon epithelial cells and colorectal cancer.

## Data Availability

The data presented in this study are available on request from the corresponding author (K.-J.R.; kjrhee@yonsei.ac.kr).

## Supplementary Materials

The following are available online at https://www.mdpi.com/article/10.3390/biomedicines10040827/s1, Table S1: Antibodies used in this study.
