# Supplementary material for: Bacteroides fragilis Toxin Induces Intestinal Epithelial Cell Secretion of Interleukin-8 by the E-Cadherin/β-Catenin/NF-κB Dependent Pathway
Source: Biomedicines. 2022 Mar 31;10(4):827. doi: 10.3390/biomedicines10040827 (PMC9032310; doi:10.3390/biomedicines10040827)
Supplement: Supplementary file 1 [file biomedicines-10-00827-s001.zip › biomedicines-1647548-supplementary.pdf]

Bacteroides fragilis toxin induces intestinal epithelial cell secretion of interleukin-8 by E-cadherin/β-catenin/NF-κB dependent pathway

Chang-Gun Lee, Soonjae Hwang, Sun-Yeong Gwon, Chanoh Park, Minjeong Jo, Ju-Eun Hong and Ki-Jong Rhee*

Supplementary Table S1. Antibidies used in this study.

Target protein (MW)	Clone #	Dilution	Company (Cat #)
E-cadherin (120 kDa)	C36	1:1000	BD biosciences (#610181)
β-catenin (92 kDa)		1:1000	Cell signaling (#9562)
NF-κB p65 (65 kDa)	D14E12	1:1000	Cell signaling (#8242)
p38 (38 kDa)		1:1000	Cell signaling (#9212)
phospho-p38 (38 kDa)	D3F9	1:1000	Cell signaling (#4511)
ERK (44, 42 kDa)	137F5	1:1000	Cell signaling (#4695)
phospho-ERK (44, 42 kDa)	D13.14.4E	1:1000	Cell signaling (#4370)
Lamin B_1 (66 kDa)	B-10	1:1000	Santa Cruz (#sc-374015)
α-tubulin (55 kDa)	B-7	1:1000	Santa Cruz (#sc-5286)
GAPDH (36 kDa)	6C5	1:1000	Sigma (#CB1001)
Occludin (59 kDa)		1:1000	Zymed (#71-1500)
Claudin 4 (22 kDa)	3E2C1	1:1000	Invitrogen (#32-9400)
p120 (120 kDa)	6H11	1:1000	Santa Cruz (sc-23873)
Peroxidase goat anti-mouse IgG		1:10000	Jackson Immunoresearch (#115-035-003)
Peroxidase goat anti-rabbit IgG		1:10000	Jackson Immunoresearch (#111-035-003)
